# Biochar for Soil Amendment: Applications, Benefits, and Environmental Impacts

**DOI:** 10.3390/bioengineering12111137

**Published:** 2025-10-22

**Authors:** Ujjwal Pokharel, Gururaj Neelgund, Ram L. Ray, Venkatesh Balan, Sandeep Kumar

**Affiliations:** 1 Department of Civil and Environmental Engineering, Old Dominion University, 135 Kaufman Hall, Norfolk, VA 23529, USA; upokh001@odu.edu; 2 College of Agriculture, Food and Natural Resources, Prairie View A&M University, Prairie View, TX 77446, USAraray@pvamu.edu (R.L.R.); 3 Department of Engineering Technology, Cullen College of Engineering, University of Houston, Sugar Land, TX 77479, USA; vbalan@central.uh.edu

**Keywords:** biochar, engineered biochar, soil amendment, carbon sequestration, pyrolysis, biomass

## Abstract

The excessive use of chemical fertilizers results in environmental issues, including loss of soil fertility, eutrophication, increased soil acidity, alterations in soil characteristics, and disrupted plant–microbe symbiosis. Here, we synthesize recent studies available from up to 2025, focusing on engineered biochar and its application in addressing issues of soil nutrient imbalance, soil pollution from inorganic and organic pollutants, soil acidification, salinity, and greenhouse gas emissions from fields. Application of engineered biochar enhanced the removal of Cr (VI), Cd^2+^, Ni^2+^, Zn^2+^, Hg^2+^, and Eu^3+^ by 85%, 73%, 57.2%, 12.7%, 99.3%, and 99.2%, respectively, while Cu^2+^ and V^5+^ removal increased by 4 and 39.9 times. Adsorption capacities for Sb^5+^, Tl^+^, and F^−^ were 237.53, 1123, and 83.05 mg g^−1^, respectively, and the optimal proportion of polycyclic aromatic hydrocarbon (PAH) removal was 57%. Herbicides such as imazapyr were reduced by 23% and 78%. Low-temperature pyrolyzed biochar showed high cation exchange capacity (CEC) resulting from improved surface functional groups. Although biochar application led to a yield increase of 43.3%, the biochar–compost mix enhanced it by 155%. The analysis demonstrates the need for future studies on the cost-effectiveness of biochar post-processing, large-scale biochar aging studies, re-application impact, and studies on biochar–compost or biochar–fertilizer mix productivity.

## 1. Introduction

Biochar, produced by pyrolyzing biomass with limited oxygen, efficiently adsorbs metal contaminants and improves soil conditions [1]. It has been used for over 2000 years to enhance soil fertility (as in Terra preta soils), and provides a simple and sustainable method for improving soil health with minimal expertise [2].

Biochar is produced using reactors such as fixed-bed, fluidized-bed, rotary kiln, or batch systems; each reactor type is appropriate for different scales of operation and feedstocks (see Figure S1). Typical feedstocks comprise agricultural residues, wood chips, pellets, and briquettes. Loose substrates need minimal processing, while densified forms allow for greater control and yield during pyrolysis. Some of the common biochar production methods include slow pyrolysis conducted at low to moderate temperatures (350–450 °C) with a residence time of 5–30 min; fast pyrolysis involves high temperatures (400–600 °C) with a residence time of 1 s; gasification is performed at temperatures of 700–1000 °C with a residence time of 10–20 s; and hydrothermal carbonization is a low-temperature and high-pressure process that uses water as the reaction medium (200–350 °C) [3]. Common energy sources include using part of the biomass feedstock as fuel for heat, electrical heaters, natural gas or propane, hydrogen, solar concentrators, and heat produced during pyrolysis. The choice of energy source influences production efficiency, cost, and sustainability of the process.

Higher temperatures and longer residence times favor gas production, while lower temperatures promote char formation. Slow pyrolysis yields the highest amount of biochar, while rapid pyrolysis maximizes bio-oil production, and gasification produces the most syngas [4]. Slow pyrolysis produces larger biochar particles with lower tar content, making it more suitable for soil amendment, whereas fast pyrolysis results in smaller, fragmented biochar with higher tar content [5].

This review examines biochar as a soil amendment. A bibliometric analysis of 5792 articles (2007–2024) indicates that “biochar”, “soil”, “amendment”, and “heavy metal analysis” are among the most frequently studied topics. Cadmium (Cd) is the most studied heavy metal, and polycyclic aromatic hydrocarbons (PAHs) are the main organic compounds investigated for soil improvement. This shows the persisting widespread issues caused by cadmium contamination in soil. Additionally, management of PAHs needs to be addressed to reduce toxicity in the environment. Despite different studies on biochar and its application in the soil, comprehensive synthesis and concrete results of recent biochar studies about its application in soil remain lacking. This paper critically analyzes all avenues of biochar from recent studies such as soil amendment, including raw material selection, production methods, modification techniques, physicochemical properties, agronomic and environmental benefits along with the risks of application, its techno–economic and life cycle analysis, and sustainability, thereby identifying research gaps and offering directions for future studies.

## 2. Biochar Production

Pyrolysis conditions, such as residence time, temperature, and pressure, directly affect biochar yield and characteristics. Biochar is derived from various feedstocks, including agricultural, dairy, paper, poultry, animal, human, kitchen, and industrial waste. Table 1 summarizes changes in biochar properties with different feedstocks and changes in pyrolysis conditions. Higher pyrolysis temperatures reduce biochar yield by shifting carbon to gas and bio-oil, while increasing ash concentration in the residue.

Biochar from woody biomass contains more carbon and has a higher C/N ratio than non-woody sources. Manure-based biochar, with its lower C/N ratio, affects nitrogen immobilization in soils [6]. Research indicates that the use of biochar can reduce nitrogen fertilizer requirements due to improved nitrogen bioavailability [7]. Table 1 shows that the carbon content of biochar increases with higher pyrolysis temperatures, as well as during field aging. One study observed a 40% increase in carbon content over a four-year period [8]. Meanwhile, oxygen (O) and hydrogen (H) contents decrease as temperature increases, due to the breakdown of oxygenated bonds.

**Figure 1:**
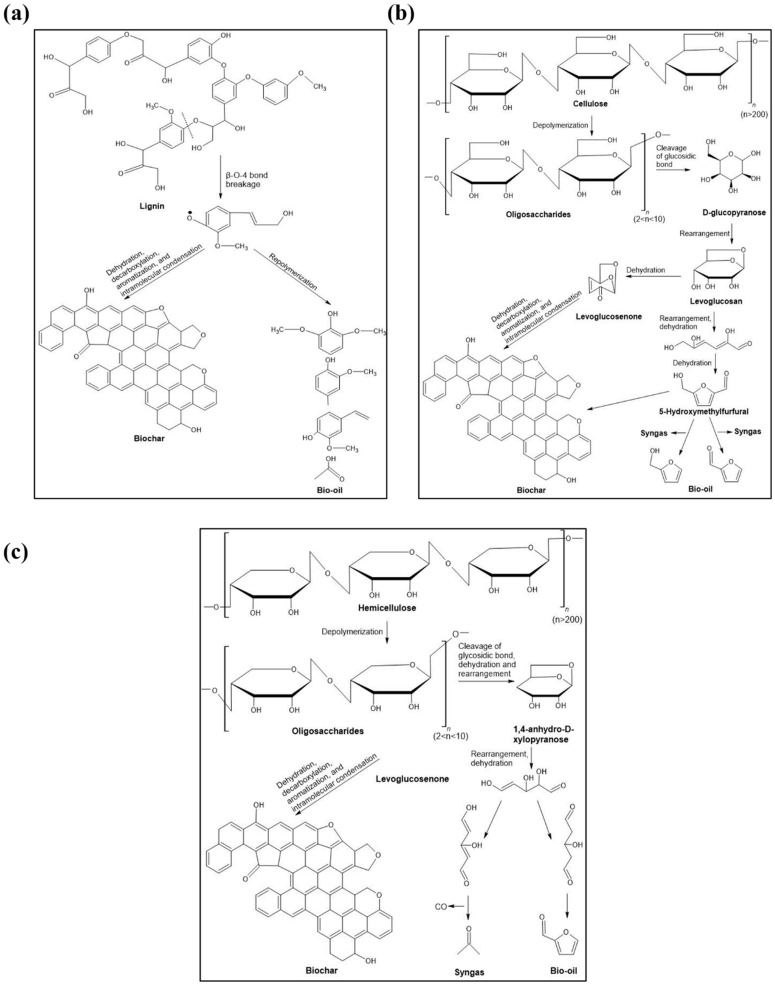
Decomposition of lignocellulosic biomass components during pyrolysis: (**a**) lignin; (**b**) cellulose; and (**c**) hemicellulose. Source: [10].

**Figure 2:**
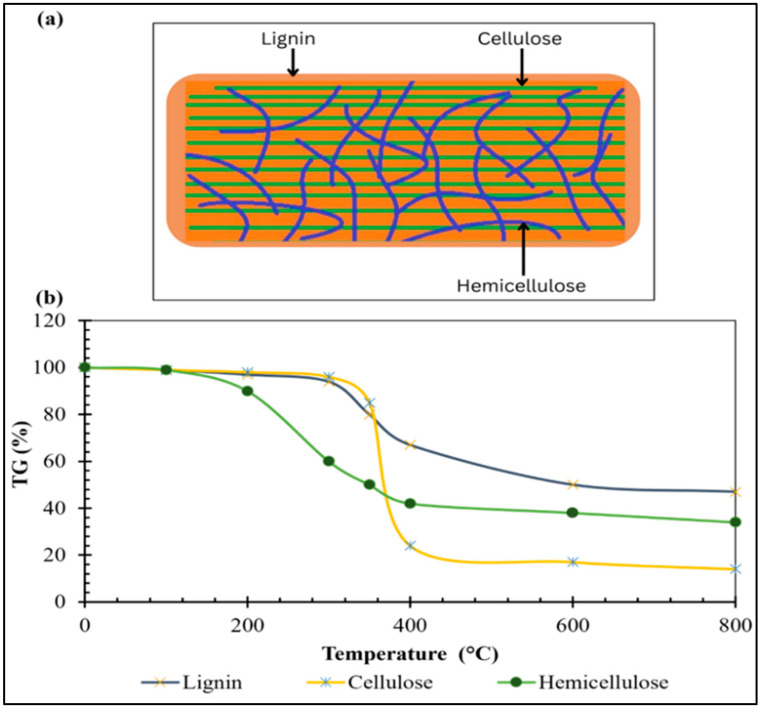
Composition of lignocellulosic biomass and its degradation profile. Here, (**a**) ultrastructure of lignocellulosic biomass comprises components such as lignin, cellulose, and hemicellulose. The orange matrix indicates lignin, green horizontal line represents cellulose, and purple/blue curved lines depict hemicellulose. (**b**) Representative figure of lignocellulosic biomass components’ decomposition against temperature measured using thermogravimetric analysis (TGA).

Higher temperatures also accelerate the breakdown of hemicellulose, cellulose, and lignin into volatile gases, further reducing oxygen and hydrogen content while eliminating polar functional groups (Figure 1). However, the decrease in functional groups reduces the CEC of a biochar. The H/C and O/C ratios are indicators of biochar stability and carbon sequestration potential. It decreases with increasing temperature and signifies enhanced stability [9]. The decomposition of hemicellulose, cellulose, and lignin occurs at different temperature ranges, with hemicellulose breaking down at 220–315 °C, cellulose at 315–400 °C, and lignin at 160–900 °C, hence altering the properties of biochar at different pyrolysis temperatures [10]. Figure 2 illustrates this concept.

**Table 1:** Effect of pyrolysis temperature on characteristics of biochar.

Feedstock	Pyrolysis Temperature (°C)	Yield (%)	C (%)	N (%)	H (%)	S (%)	O (%)	Ash (%)	Reference
Coconut husk	500	45.0	79.8	0.4	2.2	0.1	7.4	10.1	[11]
Orange bagasse	500	34.0	72.1	2.6	1.8	0.1	7.3	16.1	[11]
Peanut shell	300	36.9	68.3	1.9	3.9	0.1	25.9	1.2	[12]
	550	-	67.4	1.3	29.2	-	11.6	6.7	[13]
	700	21.9	83.8	1.1	1.8	0	13.3	8.9	[12]
Pig manure	300–700	63.0–42.8	-	2.9–6.1	-	-	-	-	[14]
Pine wood	500	30.0	88.2	0.5	2.7	0.1	6.1	2.5	[11]
Pine wood	300–700	45.5–23.2	-	0.1–0.9	-	-	-	0.4	[14]
Rice straw	300–700	45.2–30.6	69.6–81.1	0.1–0.9	-	-	-	-	[14]
Sewage sludge	300–700	-	-	6.1–0.9	-	-	-	-	[14]
Sorghum bagasse	350	38.9	62.6	-	-	-	13.1	-	[15]
	700	27.1	75.8	-	-	-	0.8	-	[15]
Soybean stover	300	37.0	68.8	1.9	4.3	0	25.0	10.4	[12]
	700	21.9	83.8	1.1	1.8	0	13.3	8.9	
Wheat straw	300	35.9	53.1	0.9	3.7	0.7	23.9	17.7	[16]
	500	26.7	55.7	0.9	2.0	0.9	16.6	24.0	
	700	23.9	57.7	0.7	1.2	0.8	7.9	31.7	
Wood	450	-	82.7	0.5	2.9	-	8.3	3.0	[17]

The decomposition process during pyrolysis releases volatile compounds such as light hydrocarbons (ethane, ethylene, propane, propylene, butane, butene); oxygenated compounds such as acids (acetic/formic acid); aldehydes (formaldehyde, acetaldehyde); ketones (acetone, hydroxy acetone); alcohols (methanol, ethanol); furans (furfural, hydroxy-methyl furfural); phenols (cresols, guaiacol); aromatic compounds (benzene, toluene, xylene); nitrogenous compounds (ammonia, hydrogen cyanide); and sulfurous compounds (hydrogen sulfide, thiols) which can be condensed, leaving behind carbon-rich biochar.

Biomass type and pyrolysis conditions influence the release of volatile compounds. All biomasses have distinct lignocellulosic contents. The variation in pyrolysis temperature affects the rate of devolatilization and decomposition of these compounds, resulting in differences in volatile compound release [18]. High-pyrolysis biochar are composed of stable compounds like pyranones, ethers, and quinine, while low-pyrolyzed biochar still has some volatile fractions like hydroxyl, carbonyl, carboxyl, and hemiacetal left, with most of the volatiles being released during the reaction [19]. Additionally, the quantity of the volatile matter increases with longer holding time, as a result of carbon released as carbonaceous gases [20]. Biochar produced at higher temperatures show increased ash content and pH from oxides and carbonates [21]; its hydrophobicity, caused by non-polar compounds and aromatic structures, needs further investigation.

## 3. Biochar Engineering

Biochar is often modified chemically or physically during or after pyrolysis to improve its properties for applications such as soil amendment and pollutant removal [22]. It adjusts soil pH to improve the availability of important nutrients like nitrogen, phosphorus, potassium, calcium, and magnesium, which help to boost plant growth [23]. In addition, it enhances moisture retention, increases cation exchange capacity, supports microbial diversity, improves soil porosity, and helps retain nutrients and organic matter for higher crop yields. Engineered biochar helps offset human-caused soil contamination and restore fertility. It lowers soil electrical conductivity, bulk density, boosts strength [24], and affects soil temperature and thermal properties [25]. The results of physical and chemical modification are provided in Table 2.

### 3.1. Chemical Method

Biochar is carbonized and activated to add functional groups, and chemical modifications further enhance its porosity, surface area, CEC, pH, and reactivity. Common acids used in biochar treatment, such as hydrochloric, sulfuric, nitric, phosphoric, oxalic, and citric acids [26] effectively remove pyrolysis impurities and lower the ash content. Alkaline treatments with sodium or potassium hydroxide improve biochar’s surface functional groups [27,28]. Agents like hydrogen peroxide (H_2_O_2_), potassium permanganate (KMnO_4_), ozone (O_3_), and ammonium persulfate (NH_4_)_2_S_2_O_8_ oxidize surfaces, adding oxygen-containing groups [29,30,31].

Ammonia (NH_4_) and urea introduce nitrogen functionalities into biochar [32]. Diversifying functional groups increases nutrient adsorption in soil. Al-modified biochar enhances NO_3_^−^ and PO_4_^3−^ adsorption, while CO_2_ and Mg/Al modifications further improve NO_3_^−^ uptake via surface charge effects [33,34].

Biochar treated with FeCl_3_, HCl, HNO_3_, NaOH, and H_2_O_2_ is also effective for NH_4_^+^ adsorption [35,36]. Engineered biochar helps reduce nutrient loss and remove contaminants, resulting in improved crop yields. NH_4_, HNO_3_, H_2_O_2_, and KOH treatments immobilize lead, while K and CuO impregnation retain other contaminants. Modifications with Na_2_S, amine, or thiol groups increase Hg^+^ adsorption. H_3_PO_4_, HCl, KOH, and ZnCl_2_ are used to remove Cr^4+^; citric acid, KOH, and H_3_PO_4_ are effective for dye removal [37,38,39], while KMnO_4_, MnO_2_, Fe, and Zn are used to eliminate Cd^2+^, Pb^2+^, Cu^2+^, and As^5+^ [40,41,42,43].

### 3.2. Physical Method

Physical modification of biochar uses steam treatment, gas purging, and UV exposure to improve surface area and porosity. Steam treatment is particularly effective for increasing surface area, removing volatile organics, and boosting soil pH and CEC [44]. Gas purging with CO_2_ or NH_4_^+^ enhances biochar pore formation and surface area [45,46]. UV-treated biochar enhances surface area and oxygen groups for improved contaminant removal [47], while magnetized rice straw biochar increases tetracycline extraction [48], and ultrasound-assisted NaOH treatment further boosts Cu^2+^ adsorption [49]. Carbon nanotube–biochar increases surface area by 97% and effectively removes dyes [50], while graphene–biochar composites enhance phenol and methyl blue adsorption [51,52].

**Table 2:** Summary of the effects of chemical and physical modification of biochar.

Raw Material	Biochar Modification	Plant Studied/Active Matrix	Result	References
Chemical modification				
Peanut Shell	P	*Pseudostellaria heterophlla*/ Soil	Increase in Cd^2+^ removal by 73%phos, root length density by 61.1%, and yield by up to 301%.	[53]
Peanut Shell	MgO	Rice plant/ Soil	Increase in PO_4_^3−^ adsorption by 20%, rice biomass by 8%.	[54]
	Sulfur–iron	Soil	Cd^2+^ removal up to 29.71%, increased bacterial abundance.	[55]
	Fe	Soil	Atrazine reduced at a rate of 100 mg L^−1^ and bacterial diversity was well maintained in contaminated soil.	[56]
Pine needle	Sulfur	Water	Hg^2+^ adsorption was 0.349 g mg^−1^ min^−1^.	[57]
Oil Palm dry bunches	Chitosan	Soil	Herbicide imazapic adsorption increased by 23%; imazapyr enhanced by 78%.	[58]
Rice husk	Chitosan	Soil	Imazapic adsorption increased by 11%, and imazapyr enhanced by 31%.	[58]
Physical modification				
Coconut shell	HCl and ultrasonication	Soil	Cd^2+^, Ni^2+^, Zn^2+^ removal efficiency of 30.1%, 57.2%, and 12.7%, respectively. The bacterial community increased by 150%.	[59]
Wood	UV irradiation	NA	Adsorption of toluene increased from 12.80 mg g^−1^ to 54.60 mg g^−1^.	[60]
Microalgae	Steam activation	Water	Adsorption of Cu^2+^ by steam activation increased by 4-folds compared to the KOH-modified biochar..	[61]
Bagasse	Ball milling	Water	Ni^2+^ adsorption increased by 6-folds compared to unmodified biochar.	[62]
Wheat straw (WS), coconut (CS), willow (WS)	Steam activation	Soil	PAHs reduced in WS, CS, and WS by 57%, 48%, and 47%, respectively.	[63]

All results are compared with the performance of pristine biochar.

## 4. Characterization Methods

**Figure 3:**
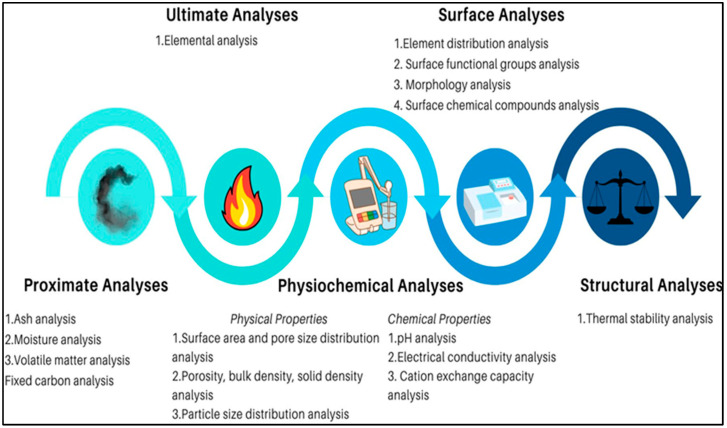
Methods and instruments used for biochar characterization.

Biochar is characterized using analytical techniques that assess its chemical, physical, and structural properties (Figure 3) [64]. The International Biochar Initiative (IBI) categorized biochar into three groups for testing based on the impact on soil functions and toxicity levels [65]. Category A measures basic characteristics of a biochar based on proximate analyses and physicochemical characteristics while category B specifies the maximum threshold for toxicants in a biochar such as PAHs, dioxins/furans, PCBs, and heavy metals. Testing of categories A and B is mandatory for all biochar. Category C includes advanced characteristics such as total surface area, mineral content, and volatile matter. Specific methods and standards are adopted to characterize the biochar. Table 3 provides the summary of standards and methods used in the analysis of biochar adopted from IBI [65].

The proximate analysis measures ash, moisture, volatile matter, and fixed carbon. The fixed carbon in a biochar is calculated using the equation 100% − (Moisture% + Ash% + Volatile matter%). On the other hand, ultimate analysis measures elemental compositions, such as carbon, nitrogen, hydrogen, sulfur, and oxygen. Dulong’s equation is utilized to calculate the calorific value of a biochar. Additionally, a chemical formula for the produced biochar can be formulated using this analytical technique. Metal content in a biochar is evaluated using ICP-MS or ICP-OES. ICP-OES uses plasma technology to excite the atoms and measure heavy metal concentration. ICP-OES allows concentration measurements of 74 different elements in the periodic table. Physicochemical analysis uses the BET method to measure the biochar’s surface area along with pore volume, pore size, and therefore it is essential to know the microporous or mesoporous properties of it. N_2_ is the adsorbate, while He is used as the carrier gas in the process. Similarly, meters such as pH and an electrical conductivity meter are also utilized. Surface analysis employs SEM-EDX, which gives high resolution picture of the surface of the material, while EDX shows the chemical composition of the surface. Furthermore, FTIR is employed to understand the functional groups present on the surface, while Raman Spectroscopy provides specific chemical fingerprints that identify material composition. Similarly, Boehm Titration, and XPS are beneficial for elemental composition, functional group identification, and morphology determination. TGA evaluates the structural and thermal stability of a biochar.

## 5. Application of Biochar

Biochar improves agricultural production by enhancing soil pH, moisture, microbial diversity, porosity, and nutrient retention, while lowering electrical conductivity, bulk density, and strength. Its porous structure also boosts nutrient availability.

Biochar has demonstrated the potential to enhance crop yields in agriculture. For instance, applying 1 kg m^−2^ of biochar increased durum wheat yields by 10% and maize yields by 6%, with a further 24% boost when combined with maize residues [74]. In perennial ryegrass pot experiments, a 6 kg m^−2^ application rate raised dry matter by 120%, though higher amounts reduced production, highlighting the importance of optimal rates for application [75]. Biochar is a pathway to long-term carbon sequestration, thus supporting sustainable agriculture [76]. Table 4 summarizes these effects on soil.

### 5.1. Bulk Density and Porosity

Bulk density is an indicator of soil compaction, determined by the ratio of the dry weight of soil and its total volume, indicating pore spaces. Porosity refers to the volume of these pore spaces in the soil, which affects water retention and root growth [86]. Porosity is calculated using Equation (1), where “O_b_” represents bulk density and “O_d_” represents particle density.

(1) $$\varepsilon =1\;-\;\frac{O_{b}}{O_{d}}$$

Biochar produced at 400 °C demonstrated a bulk density reduction of approximately 20% when applied to loamy sand [79]. However, it increased soil porosity by up to 18% in sandy and sandy loam soils, depending on the biochar application rate [78]. Similar improvements in porosity were observed with biochar made from corn residues at the same temperature, with higher application rates correlating with greater porosity [87].

### 5.2. Tensile Strength and Particle Density

Tensile strength is the maximum tension a soil can withstand before breaking. Lower tensile strength usually means lower density, less resistance to penetration, and greater porosity and water retention. Research indicates that higher biochar application rates can completely reduce tensile strength in clay soils [88]. Particle density, defined as soil mass per unit volume (excluding voids and water), is influenced by biochar. For example, applying 3 kg m^−2^ of wood biochar decreased particle density by 13.7% in arable land but showed no notable effect on grassland [25]. More field studies will clarify biochar’s definitive impact.

### 5.3. Water Repellency

Soil water repellency describes soil’s hydrophobicity, typically assessed by the Water Drop Penetration Time (WDPT) method, which measures how long water takes to absorb into the soil surface. Biochar, particularly in its outer layers, also exhibits hydrophobic characteristics [89]. However, biochar produced at higher temperatures, such as corn biochar at 750 °C or orchard pruning biochar at 500 °C, exhibited minimal water repellency. Some studies even report decreased soil hydrophobicity following biochar application [82]. While lower-temperature biochar tends to be more hydrophobic, increased hydrophobicity has also been noted at higher pyrolysis temperatures [90]. Interestingly, biochar alone often shows greater water repellency compared to biochar mixed with soil [91]. Given the limited research on biochar’s water-repellent properties, further investigation will clarify its effects.

### 5.4. pH Change

Biochar releases alkaline substances that lower soil acidity, and its negative charge helps retain cations, stabilizing soil pH [83]. Application of biochar can reduce soil acidity by over 50% [92]. However, too much alkalinity can restrict plant uptake of micronutrients such as Fe^2+^/Fe^3+^, Zn^2+^, Mn^2+^, and Cu^2+^; for example, Fe^2+^/Fe^3+^ absorption declines in alkaline conditions, deteriorating plant health [93]. Therefore, while biochar regulates soil pH and boosts yields, limits in dosage are required to prevent excessive alkalinity.

### 5.5. Cation Exchange Capacity

**Figure 4:**
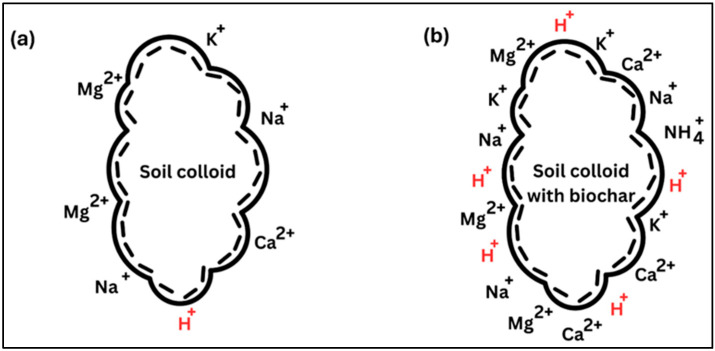
CEC of soil with and without biochar. (**a**) Low-CEC soil colloid without biochar. (**b**) High-CEC soil colloid after the integration of biochar.

Cation exchange capacity (CEC) is a key soil property that affects nutrient availability and plant growth [94]. Mainly driven by organic matter and clay in biochar [95], CEC measures the soil’s total negative charge, allowing it to retain cationic nutrients like H^+^, Ca^2+^, Mg^2+^, Na^+^, and NH_4_^+^, and thus minimize leaching [96]. This improves soil fertility, buffering, and water retention [97], hence supporting sustainable plant growth. Biochar’s CEC is influenced by negatively charged sites, such as deprotonated oxygen-containing groups, which exchange with positively charged ions [98]. Figure 4 illustrates It demonstrates the increased affinity of H^+^, K^+^, Ca^2+^, NH_4_^+^, Na^+^, and other metallic ions after biochar application and, Table 5 summarizes the effect of temperature on biochar’s CEC across various feedstocks. 

Biochar’s CEC tends to increase at low to medium pyrolysis temperatures before peaking at around 500 °C [101]. However, CEC can fluctuate depending on the biochar application rate and feedstock used. 

### 5.6. Organic Pollutants

Biochar is well known for pesticide and herbicide adsorption, demonstrating its ability to limit contaminants leaching into groundwater and cause adverse effects on human and aquatic health. Biochar’s porosity plays a crucial role in capturing contaminants from soil. Porosity of the biochar is an important factor for capturing the adsorbed contaminants from the soil [102,103]. Various biochar types, including those derived from rice straw, softwood, coconut shell, and bamboo, have shown significant adsorption potential for herbicides like fomesafen [104].

Biochar made from pecan and hickory wood effectively adsorbs herbicides like clomazone and bispyribac sodium, but desorption rates rise after repeated use [105]. Biochar produced from oil palm, rice husk, maize stover, switchgrass, and woodchips was found to decrease the bioavailability of herbicides such as imazapyr, atrazine, terbuthylazine, imidacloprid, thiamethoxam, and diuron, along with other organic pollutants in soil [106,107,108,109,110]. Similarly, atrazine and nicosulfuron exhibited high affinity to peanut shell biochar [111]. Cotton straw and woodchip biochar can reduce pesticides like carbofuran, chlorpyrifos, and fipronil [112,113,114]. However, the use of engineered biochar further enhanced the removal of organic pollutants and diversified the affinity to a variety of harmful organic compounds in the soil. For instance, iron-modified biochar was effective in removing chlorpyrifos [115], while nitrogen doping and sulfuric acid treatment increased the adsorption capacity of atrazine [116,117]. In addition, biologically modified biochar effectively removes paraquat [118]. Its use in the agricultural field requires proper planning as it limits the effectiveness of pesticides and herbicides against pest and weed control, limiting agricultural productivity. Future studies should focus on refining biochar and pyrolysis methods to balance pollutant removal with pesticide effectiveness.

### 5.7. Inorganic Pollutants

Biochar helps to prevent harmful metals from contaminating soil and groundwater, lowering their bioavailability and plant toxicity. Biochar types like rice straw, tomato waste, and sugarcane bagasse have shown effective heavy metal removal from soil, with Cd^2+^ removal rates of 73%, 34%, and 63% at respective application [119]. Sugarcane bagasse biochar achieved 85% Cr^4+^ removal [120], while rice straw biochar was effective for Pb^2+^ and Cu^2+^, especially at higher application rates [121].

Engineered biochar infused with transition metals and oxides demonstrates higher removal efficiency; for example, Fe^2+^/Fe^3+^ corn stalk biochar adsorbed 170 mg g^−1^ of Cd^2+^, while K_2_FeO_4_^−^ treated biochar reached 80 mg g^−1^ [122,123]. Reduced metal bioavailability results from mechanisms such as complexation, cation exchange, and electrostatic interactions [124]. The efficiency of biochar in remediating specific metals depends on feedstock; for instance, pecan shell biochar works well for Ni^2+^ and Cd^2+^, while kitchen waste biochar is effective for Cd^2+^ and Pb^2+^ [125,126]. Ferrous sulfate engineered biochar showed a 39.9 times and 3.7 times decrease in both water-soluble and bioavailable V^5+^ in the soil [127]. Reducing Sb^5+^ contamination in rice fields is the only option to reduce human intake of antimony [128]. MnFe_2_O_4_ modified biochar showed optimum adsorption of 237.53 mg g^−1^ of Sb^5+^. However, studies have also shown an increase in Sb^5+^ content in the plant shoot after biochar application [129]. Similarly, magnetite-modified biochar removed thallium (Tl^+^) at a rate of 1123 mg g^−1^, sulfur-modified rice husk biochar removed Hg^2+^ by 99.3%, MgO-modified BC showed 83.05 mg g^−1^ fluoride (F^−^) removal, rare earth material europium (Eu^3+^) removal rate was 99.2% [130], and samarium (Sm^3+^) maximum uptake was 350 mg g^−1^ [131]. Similarly, other rare earth materials like cerium (Ce^3+^) and neodymium (Nd^3+^) have shown a high affinity to biochar [132,133]. Thus, appropriate raw materials, production, and modification methods are essential to optimize inorganic pollutant uptake for soil remediation.

### 5.8. Microbial Communities

Biochar improves soil qualities such as pH, toxicity, carbon content, and CEC, creating better conditions for soil microbes. Its porous structure shelters microbial communities, while the labile carbon and water serves as a source of food boosting their survival and longevity [134]. Although certain groups of microbes thrive in the presence of food, some microbial groups are impacted by its toxicity, but the impact is minimal [135]. Research indicates that biochar increases microbial biomass [136]. Biochar with larger macropores (>15 µm) benefits soil microbes more than biochar with smaller micropores, as larger pores offer greater specific surface area. Thus, the temperature and biomass type for biochar production play a significant role in microbial diversity [8]. On the other hand, aging reduces nutrients and porosity, proving detrimental to microbial populations [137].

pH is another factor that affects the diversity and population of microbial communities. A basic biochar positively benefits the relative abundance of the microbial community [138]. Nitrifying and soil nutrient mineralizing bacteria populations depend on soil pH [139]. Biochar impact on microbial diversity is even more pronounced in clay soils, although the effects vary based on soil type, biochar feedstock, toxic chemicals, nutrient availability, and vegetation [140]. For instance, rice straw biochar increased the biomass of *Ascomycota* and *Chytridiomycota*, while wood-derived biochar tends to reduce it [141,142].

**Figure 5:**
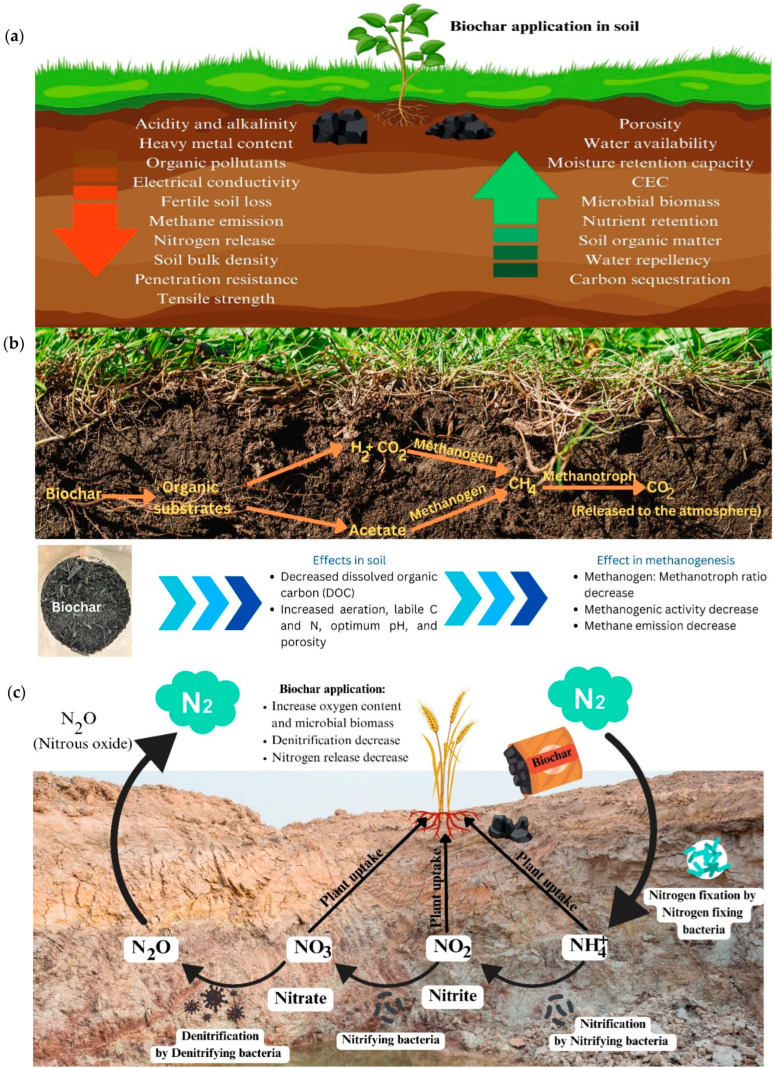
Shows how biochar helps reduce methane emissions, highlighting its value for sustainable soil management. (**a**) Impact of biochar as soil amendment. The downward arrow indicates a decrease, and upward arrow indicates an increase in the corresponding soil properties. (**b**) Role of biochar in methane emission reduction. (**c**) Impact of biochar on nitrous oxide emission reduction through diversification of microbial community.

However, approximately one-third of studies from 2010 to 2022 reported negative effects of biochar on microbial populations [143]. For example, applying 2% and 4% pine biochar decreased phosphorus availability and reduced arbuscular mycorrhizal fungi (AMF) in plant roots [144]. Conversely, biochar can increase available phosphorus and alter AMF populations, as demonstrated with mango biochar application [145]. Biochar takes part in redox reactions such as nitrification and denitrification because of redox-active moieties that play a role in greenhouse gas emissions. Figure 5 highlights the diversification of microbial communities and their impact on nitrous oxide emissions.

In short, engineered biochar regulates the soil pH and promotes the growth of a specific microbial population, further improving soil quality.

### 5.9. Carbon Sequestration

Biochar helps to mitigate climate change by effectively sequestering carbon, stabilizing about 50% of the carbon in biomass. It also improves soil health and boosts crop yields, but its main value lies in long-term carbon storage, outperforming burning or direct land application. Biochar reduces greenhouse gas emissions, improves soil fertility and crop yields, and provides long-term carbon storage due to its stability [146]. Its porous structure and minerals support soil biology and enhance carbon retention, contributing to climate change mitigation [147].

## 6. Techno–Economic Analysis

Techno–economic assessment (TEA) evaluates the technical and economic performance of a product. It analyzes the cost, profits, risks, and uncertainties, and demonstrates its economic feasibility [148]. Different studies employing TEA of biochar are presented in Table 6. Currently, the biochar market is still in the developmental stage, with minimal revenue generation posing challenges to its commercialization. Inefficiencies in transportation and recyclability have added setbacks to the growth of the market. The issues, such as disintegration and dust emission, need consideration to enhance its value in the market. Granulation and palletization might be potential techniques to address the issue of durability and recyclability, thus adding value to the product.

The minimum selling price (MSP) of biochar is highly dependent on the location of production, biochar yield, conversion technology, and carbon sequestration subsidy [149]. A portable pyrolysis unit eliminates the need for transportation and packaging costs, making it more affordable [150,151]. A biorefinery producing biochar with other products can help to reduce production costs [152]. Syngas byproducts can be converted into methanol for additional revenue [149]. Hence, the way forward to reduce the selling price of the biochar is to establish an integrated biorefinery that produces biochar along with other biofuels. Carbon offset incentive is another alternative to reduce the price. Biochar prices range from USD 0.012 to USD 0.100 per kg, depending on the level of carbon offset incentive [153]. Pyrolysis techniques such as flame curtain pyrolysis, rotary cavity kiln, double-chamber kilns, top-lift updraft gasifiers, anila stoves, retort kiln, and earth-covered out kiln can reduce the cost for biochar production significantly. These pyrolysis processes can be used in a rural setting, as the biochar production is inexpensive, with an added incentive of heat for cooking. Although these techniques are simple to operate, complete pyrolysis takes a longer time, sometimes even days. Additionally, the temperature cannot be controlled, and homogeneous biochar cannot be retrieved from the system, as seen in the study with flame curtain pyrolysis [154,155]. A simple low-cost pyrolyzer demonstrated a total profit of USD 913 with biochar selling price of 2.806 per kg [156]. In contrast, modern pyrolyzers cost USD 132–200 million, produce 2000-metric-ton of biochar, and yield an IRR of 15 to 37%. The optimal biochar cost for maximum revenue ranges from USD 0.12–0.35 per kg [157,158,159], with larger production scales generating higher returns [160]. Future research may explore densification to lower transport costs and enhance profitability for the reduction in market price.

**Table 6:** Economic overview of the biochar system and product.

Feedstock	Temperature and Yield	Features	Production Capacity (kg m^−2^)	NPV/IRR/MSP	Breakeven Period	Reference
Pine	300 °C, 450 °C; Yield: 80% and 45%	Syngas converts to methanol	10	NPV: USD 0.220–0.280 kg^−1^ with 70% revenue from biochar and 30% from methanol production IRR: 14.2-10.1% (Shows moderate return)	-	[149]
Forest residues	Portable; ~680 °C–750 °C Yield: 13–21% BSI, 20% OK, 6.5% ACB	Power sources and production site distance considered	0.02–0.038	MSP for BSI is USD 3–6 kg^−1^, OK is USD 1.6 kg^−1^, and ACB is USD 0.5 kg^−1^	100 days	[151]
Grape residue	500 °C Yield: 37%	Biochar production integrated into a biorefinery	0.015	NPV: USD 111.7 million (overall biorefinery) IRR: 34.3% (Shows high return)	2.5 years	[152]
Tree pruning	450–800 °C Yield: 20.20–29.17%	Investigating the economic feasibility of biochar systems	0.121	NPV: USD 3,119,448 IRR: 22.35% (Shows high return)	8 years	[161]

## 7. Life Cycle Assessment

Life cycle assessment (LCA) evaluates biochar’s environmental effects across its life cycle, from product development to final disposal using functional units like feedstock or yield [162]. LCA scopes include cradle-to-grave (from extraction to disposal), cradle-to-gate (from harvesting to production), cradle-to-cradle, evaluating a product’s recyclability and reusability, and gate-to-gate examining manufacturing processes [163,164]. Production of biochar falls in either cradle-to-gate or cradle-to-grave approaches. Product impacts are assessed using data from Ecoinvent, and methods using ReCiPe, CML, IPCC, and IMPACT 2002+, and interpretation tools employed include OpenLCA, FaBI, and SimaPro. A variety of life cycle assessment (LCA) studies are presented in Table 7.

A biochar study on Cd^2+^ remediation demonstrated 33.73 t CO_2_ eq/ha of carbon sequestration, in addition to the reduction in Cd^2+^ to below 0.2 mg/kg in rice grains [165]. Similarly, modeling for the forest residue pyrolysis process showed the reduction of 4264 Mg CO_2_eq year^−1^ with 4800 Mg of forest residue [166]. Another study indicated the sequestration potential of 920 kg CO_2_eq year^−1^ and 0.01 kg N_2_O reduction. The same study illustrated that the biochar application process also emits 75.66–78.74 kg CO_2_eq year^−1^ during crop harvesting [167]. These studies display the role of biochar in reducing carbon footprints. Future research should target emission reduction during the production and application of biochar in the field. It should also compare the carbon footprint after application of different varieties of biochar in similar environmental conditions.

**Table 7:** Effect of biochar application on soil and LCA.

Feedstock	Pyrolysis	Methodology	Biochar Application to Soil	Impact Categories	Results	Reference
Winter oilseed rape straw	400 °C and 800 °C	IPCC 2013 manual calculation	0.1 kg m^−2^	Carbon footprint: 100 yr, 20 yr	Reduction in GHG 400 °C: 73%; 800 °C: 83%	[146,168]
Oat Waste and willow wood	-	IPCC 2013 (GaBi)	0.0025 kg–0.02 kg m^−2^	Carbon footprint: 100 yr	Reduction of 0.050 kg CO_2eq_ to 0.390 kg CO_2eq_	[168,169]
Miscanthus	Slow pyrolysis (Temperature is unknown)	IPCC 2013 (Simapro)	0.5 kg m^−2^	Carbon footprint: 100 yr	−0.737 kg CO_2eq_ kg^−1^; biochar contributes 50% carbon sink in soil	[168,170]
Tomato plant waste	Intermediate pyrolysis (Temperature 400 °C)	IPCC 2013 (Simapro)	0.1 kg m^−2^ with yield of 35%, 40% and 45%	Carbon footprint	At 80% stable C and 45% yield, kg CO_2eq_ kg^−1^ biochar is −0.156. At 20% stable, C carbon sequestration is absent	[168,171]
Paddy rice, maize	Vertical kiln at 350–500 °C	IPCC 2013	2 kg m^−2^	Carbon footprint	2.037–4.129 kg CO_2eq_ m^−2^ for paddy rice; 2.858–3.949 kg CO_2eq_ m^−2^ for maize	[168,172]
Rice straw	Top-lift (TLUD) drum oven (Temperature unknown)	IPCC 2013	0.05 kg m^−2^	Carbon footprint	610 kg CO_2eq_ in spring and 122 kg CO_2eq_ in summer	[168,173]

## 8. Optimal Biochar Application Rates

Research indicates that biochar application in the field is associated with increased photosynthesis rates and crop yields, as shown in Table 8. It is noteworthy that engineered biochar has shown better crop yields and metal removal capacity than pristine biochar in field applications. For instance, an application of 3 t ha^−1^ of attapulgite and biochar composite showed a 12.8% increase in pasture biomass in just 3 months [174]. Another study with 4.5 t ha^−1^ of biochar treated with Fe [175] and MgO [54] demonstrated increases in available phosphorus by up to 90.3% and shoot biomass by 6%, respectively. A biochar inoculated with *Pseudomonas putida* even showed an increase in fruit weight and protein weight by 7.6% and 28.6%, respectively, with 500 g biochar applied per tree [176]. A 1.5 t ha^−1^, Fe–biochar application demonstrated a reduction in As^5+^ and Cd^2+^ by 26% and 36% within 1.5 years [177]. Additionally, a 100% *v/v* biochar application led to a reduction in particle and bulk density by 39% and 18%, respectively, while increasing porosity by 56% [81].

However, several studies have reported inconsistencies between laboratory assessments and field trial outcomes due to environmental variability, uncontrolled conditions, and slow output time [178]; additionally, the majority of soil application studies only focus on laboratory conditions. Therefore, future studies must focus on large-scale and long-term field applications to examine the effect of aging and environmental conditions on the chemical and structural stabilities of biochar and field productivity.

**Table 8:** Relationship between biochar application rate and agricultural productivity.

Application Rate	Time of Application	Affected Species	Effect of Application	Reference
0%, 2%, 4%, 8% wheat straw biochar	4 months	Tomato plant	Photosynthetic rate of 17.08 ± 0.19 µmol m^−2^ s^−1^, increasing yield by 14%.	[179]
0%, 4%, 8% *Conocarpus* biochar	80 days	Tomato plant	Yield increases by 14% to 43.3%.	[180]
0–47.25 t ha^−1^	5 years	Maize	Increase in organic phosphorus by 12.8% to 66.6%.	[181]
1.6 kg m^−2^ of biochar and fertilizer	4 years	Wheat	Increase in yield by 16.3% outperforming fertilizers alone by up to 31.2%.	[182]

## 9. Integrating Biochar with Organic Composts

Studies have investigated mixing biochar with compost to improve compost properties and increase agricultural yield. Maintaining a neutral pH is essential during composting, as it supports the diversification of the microbial community, while acidic pH can stop the process. Although a 20% (*w/w*) bamboo biochar neutralized pH in poultry composting [183], it had minimal effect on swine manure pH [184]. Adding biochar reduces heavy metals and nitrogen loss, enhances aeration, organic matter breakdown, microbial activity and humic compounds, and lowers NH_4_^+^ emissions [185,186]. A biochar content of about 10% (*w/w*) is recommended for high-quality compost; higher amounts, however, lower compost quality and reduce agricultural yield [183]. It is interesting to see that a biochar–compost mixture enhances nitrogen and phosphorus uptake by plants due to greater nutrient bioavailability, and resulting in higher biomass yield [187]. A similar study using different varieties of biochar–manure mixtures for maize growth shows a higher maize yield with a low percentage of biochar, while a higher application rate limited the agricultural productivity, as shown in Table 9. Another study on corn productivity illustrated 10 t ha^−1^ as the optimum biochar application rate beyond which the productivity decreased. However, a similar study with Chinese cabbage demonstrated 15 t ha^−1^ biochar application as the optimum condition, with 10 t ha^−1^ compost also added to both studies [185,186].

As the concern today is to find an alternative to fertilizers, biochar–compost mixtures and engineered biochar both demonstrate promising results to gain maximum agricultural benefits. However, there are limited studies about compost mixed with modified biochar. Future studies must therefore focus on the use of a mixture of compost and engineered biochar for optimum agricultural production.

**Table 9:** Relationship of biochar–compost mix with agricultural productivity.

Application Rate	Plant Studied	Effect of Application	Reference
20% oak biochar-blended compost	Grape	Increase in N by 44%, K^+^ by 26%, and microbial respiration by 26%. Weight of the fruit increases by 16%.	[187]
Cow manure Biochar + Compost (5 tons each)	Maize	60% irrigation leads to an increase in yield by 107%.	[188]
9% Willow wood Biochar- compost blend	Maize	Increase in yield by 20%.	[189]
2% Grape pomace biochar–compost	Maize	Increase in biomass yield by 155%.	[190]
2% Rice husk biochar–compost	Maize	Increase in biomass yield by 5-fold.	[190]
2 t Acacia biochar in 10 t compost	Nitisol	Increase in yield by 60% and 54% in different soil groups.	[191]

## 10. Post-Processing of Biochar

Biochar is of low density and is a friable material, resulting in its highly mobile nature. It is easily erodible by air, and transports within the soil in the form of infiltration or surface runoff, which can potentially contaminate water resources. A biochar adsorbs toxic inorganic and organic pollutants, and its transport and deposition increase the toxicity level of water resources [192]. For instance, biochar colloid increased the transport rate of Cr^6+^ in the soil by seven times [193]. Additionally, in biochar produced from sewage sludge or high heavy metal (HM) biomass, there is a risk of HM and polycyclic aromatic hydrocarbon (PAH) accumulation in soils, thereby increasing toxicity [194]. A biochar is also affected by its surrounding environment, affecting its stability and thereby releasing the adsorbed pollutants for uptake by plants, which can affect the entire food chain, including human beings [195]. In addition, the inhalation of biochar dust during its production, application, or transport increases the risk of respiratory diseases [196].

**Figure 6:**
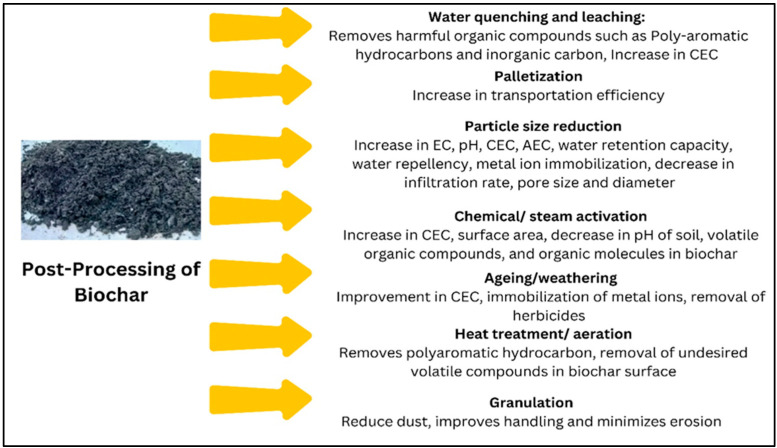
Post-processing pathways of biochar are essential for tailoring its properties to meet specific agricultural, environmental, or industrial needs.

To mitigate the issues during field application, biochar must be highly stable. Post-processing techniques maintain stability, remove impurities, improve porosity, and surface functionality in a biochar. It is often pelletized, granulated, or heat treated [197], as shown in Figure 6. The water-quenching process removes toxic compounds that can enter the soil [137]. This process lowers ash content and preserves pores, enhancing nutrient retention [198].

Biochar is compressed into pellets or granules for easier transport, with binders like starch or lignin added to increase strength and prevent erosion. Palletization cuts biochar packaging costs by about 30%, while binders like sodium carboxymethyl cellulose and starch help granulate it. These processes reduce health risks from dust exposure and reduce the health impact. Furthermore, granular bamboo biochar sells for USD 0.4–0.8 per kg in China, offering high economic returns for densified biochar. The particle size distribution of biochar plays a crucial role in determining its physical properties, including porosity, pore volume, pore diameter, bulk density, and specific surface area [199]. Some key post-processing methods are outlined. Nano-biochar, produced through ball milling and centrifugation of bulk biochar, offers transportation efficiency and cost savings due to its lower volume. While a study on biochar nanoparticles (BNPs) showed no definitive relationship between BNP application and tomato seed germination [200], another study found that BNPs produced at 300–600 °C effectively immobilized Cd^2+^ ions, enhancing rice plant growth [201]. Aging of biochar raises CEC and improves metal ion immobilization [202,203,204], while also lowering herbicide concentrations such as picloram and terbuthylazine due to increased surface oxygen groups [205]. Aging also improves ash content [196], whereas aerated heat treatment reduces volatiles and removes toxic polycyclic aromatic hydrocarbons like naphthalene [206]. The optimal post-processing method depends on specific needs, and further research is required to evaluate its cost-effectiveness.

## 11. Conclusions

The rising demand for agricultural productivity has resulted in an exponential increase in population. There are places within the globe where soil health has degraded to the extent that it cannot be used for agricultural production. Biochar alleviates the need for chemical fertilizer, addressing issues of soil nutrient imbalance, reduction in soil fertility, soil acidification, decline in microbial community, and increased salinity. The application of biochar modifies soil characteristics to enhance carbon sequestration, nutrient and water retention, pH regulation, and reduction in fertile soil loss, all of which are vital to increase agricultural productivity. Furthermore, biochar, as demonstrated in this article, improves the health of degraded soil through toxic metal and organic pollutant removal, improvement in soil properties such as bulk density, tensile strength, reduction in salinity, and enhancement in microbial activity and diversity. However, the inconsistent performance shown by biochar when produced at different conditions and feedstocks needs optimizing, and engineering a biochar using a modification method is the way forward. Future research needs to evaluate the cost-effectiveness of biochar post-processing, study the implications of biochar–compost mixtures in field application, and large-scale biochar aging. Furthermore, studies about the application of chemical fertilizer and biochar mixtures need further research to reduce fertilizer use and its negative implications to some extent.

## Figures and Tables

**Table 3:** Standard methods for biochar analysis.

Analyses Type	Parameter	Standard/Test Method	References
Proximate Analyses	Moisture Total Ash Volatile Matter	ASTM D1762-84	[66]
Chemical Analyses	pH Electrical Conductivity	TMECC (2001) and IBI	[65,67]
Physical Analyses	Particle Size Distribution	IBI	[65]
	Total Surface Area External Surface Area	ASTM D6556	[68]
Surface Analyses	PAHs	US EPA 8270 (2007) and IBI	[65,69]
	Dioxins/Furans	US EPA 8290 (2007)	[70]
	Polychlorinated Biphenyls (PCBs)	US EPA 8082 (2007) or US EPA 8275 (1996)	[71,72]
	Mercury	US EPA 7471 (2007)	[73]
	Arsenic, Cadmium, Chromium, Cobalt, Copper, Lead, Mercury, Molybdenum, Nickel, Selenium, Zinc, Boron, Chlorine, and Sodium	TMECC (2001)	[65]

**Table 4:** Summary of the effect of biochar on the properties of soil.

Property	Effect of Biochar Application	Result	Reference
Bulk density	Reduction	Decreased by up to 28%.	[25,77,78]
Porosity	Increase	Increased by up to 24%.	[79]
Tensile strength	Reduction	Decreased by up to 242%.	[80]
Particle density	Reduction	Decreased by up to 39%.	[81]
Water repellency	Regulated according to need	Low-temperature pyrolyzed biochar was more hydrophobic than high-temperature biochar.	[79,82]
pH Change	Regulated according to need	Regulated pH in the soil and increased the bioavailability of nutrients.	[83]
CEC	Increase	Low-temperature pyrolyzed biochar exhibited more CEC than high-temperature pyrolyzed biochar.	[84,85]

**Table 5:** Effect of pyrolysis temperature on the CEC of biochar.

Biochar Feedstock	Pyrolysis Temperature (°C)	CEC (cmol kg^−1^)	Reference
Douglas fir wood	350	54.0	[99]
	400	46.0	
	450	47.0	
	500	53.0	
	550	51.0	
	600	49.0	
Oak wood	400	106.0	[85]
	600	65.2	
Buckwheat husk	450	11.5	[100]
	550	10.1	
Peanut shells	450	11.1	[100]
	550	10.6	
Peat-based growing media	450	54.0	[84]
	600	11.0	
	750	8.0	
Woody green waste	450	65.0	[84]
	600	16.0	
Tree bark (*Pinus pinaster*)	450	292.0	[84]
	600	160.0	
Wheat straw	500	5.1	[101]
	600	1.3	
	700	0.5	
Corn straw	500	68.6	[101]
	600	20.1	
	700	19.0	
Peanut shell	500	8.5	[101]
	600	1.2	
	700	0.3	

## Data Availability

No new data were created or analyzed in this study. Data sharing is not applicable to this article.

## Supplementary Materials

The following supporting information can be downloaded at https://www.mdpi.com/article/10.3390/bioengineering12111137/s1, Figure S1: Biochar production method: (a) batch process and (b) continuous process.

## Abbreviations

AEC	Anion Exchange Capacity
AMF	Arbuscular Mycorrhizal Fungi
BNP	Biochar Nanoparticle
BET	Brunauer–Emmett–Teller
CEC	Cation Exchange Capacity (cmol kg^−1^)
FTIR	Fourier Transform Infrared Spectroscopy
GHG	Greenhouse Gases
ICP-MS	Inductively Coupled Plasma-Mass Spectrometry
ICP-OES	Inductively Coupled Plasma- Optical Emission Spectroscopy
IRR	Internal Rate of Return
LCA	Life Cycle Assessment
LCI	Life Cycle Inventory
MSP	Minimum Selling Price
NBC	Nitrogen-Doped Biochar
NPV	Net Present Value
PAH	Polycyclic Aromatic Hydrocarbon
PCB	Polychlorinated Biphenyl
PCDD/F	Dibenzo-P-Dioxins/Dibenzofuran
SEM-EDX	Scanning Electron Microscopy-Energy Dispersive X-Ray
TEA	Techno-Economic Assessment
TGA	Thermogravimetric Analysis
TKN	Total Kjeldahl Nitrogen
TN	Total Nitrogen
UV	Ultraviolet
XPS	X-ray Photoelectron Spectroscopy
