# Supplementary figures and images for: Biochar for Soil Amendment: Applications, Benefits, and Environmental Impacts
Source: Bioengineering (Basel). 2025 Oct 22;12(11):1137. doi: 10.3390/bioengineering12111137 (PMC12649191; doi:10.3390/bioengineering12111137)

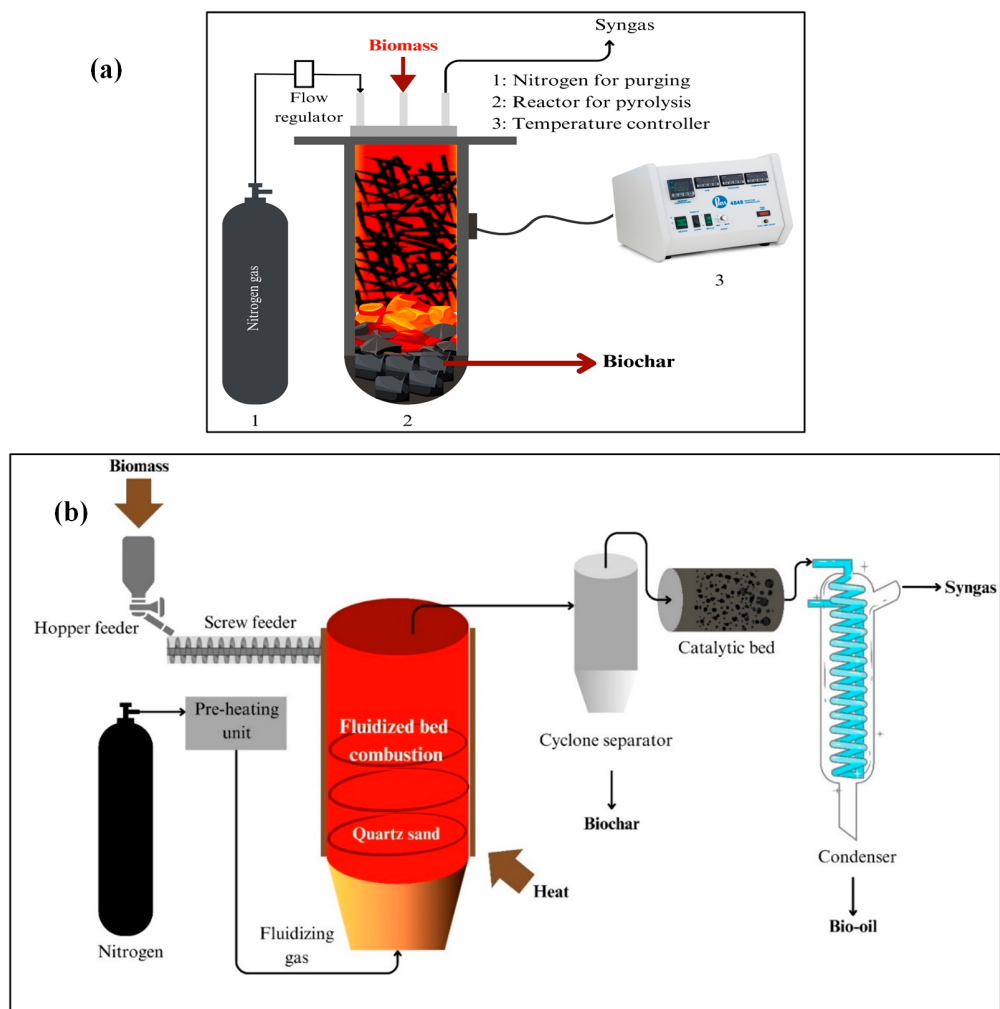

Figure S1. Biochar production method: (a) batch process, and (b) continuous process.

Supplement: Supplementary file 1 [file bioengineering-12-01137-s001.zip › bioengineering-3872360-supplementary.pdf]
